# Outcome of two pairs of monozygotic twins with pleuropulmonary blastoma: case report

**DOI:** 10.1186/s13052-020-00912-6

**Published:** 2020-10-07

**Authors:** Shihan Zhang, Xisi Wang, Sihui Li, Siyu Cai, Tong Yu, Libing Fu, Na Zhang, Xiaoxia Peng, Qi Zeng, Xiaoli Ma

**Affiliations:** 1 Beijing Key Laboratory of Pediatric Hematology Oncology, National Discipline of Pediatrics, Ministry of Education, MOE Key Laboratory of Major Diseases in Children, Hematology Oncology Center, Beijing Children’s Hospital, Capital Medical University, National Center for Children’s Health, Beijing, China; 2 Children’s Hospital of Shanxi, Women health center of Shanxi, Taiyuan, Shanxi China; 3 Center for Clinical Epidemiology and Evidence-Based Medicine, Key Laboratory of Major Diseases in Children, Ministry of Education, Beijing Children’s Hospital, Capital Medical University, National Center for Children’s Health, Beijing, China; 4 Department of Image Center, Beijing Children’s Hospital, Capital Medical University, National Center for Children’s Health, Beijing, China; 5 Department of Pathology, Beijing Children’s Hospital, Capital Medical University, National Center for Children’s Health, Beijing, China; 6 Department of Thoracic Surgery, Beijing Children’s Hospital, Capital Medical University, National Center for Children’s Health, Beijing, China

**Keywords:** Monozygotic twins, Pleuropulmonary blastoma, Long-term survival

## Abstract

**Background:**

Pleuropulmonary blastomas (PPB) are rare aggressive paediatric lung malignancies and are among the most common *DICER1*-related disorders: it is estimated that 75–80% of children with a PPB have the *DICER1* mutation. *DICER1* mutations are responsible for familial tumour susceptibility syndrome with an increased risk of tumours. In approximately 35% of families with children manifesting PPB, further malignancies may be observed. Symptoms of *DICER1* syndrome may vary, even within monozygotic twins. Preventive screening of carriers with *DICER1* mutations is important and follow-up is undertaken as recommended by the 2016 International PPB Register.

**Case presentation:**

We present two pairs of monozygotic twins. In one pair of 4-year, 2-month old girls, both with *DICER1* mutation, one developed PPB(II) and her identical sibling had acute transient hepatitis. In the other pair of 19-month-old female babies, one had a history of bronchopulmonary hypoplasia and developed PPB(III) without *DICER1* mutation, and her identical sibling had allergic asthma. Both patients with PPB were treated with R0 resection and received 12 cycles of postoperative chemotherapy. At the most recent review, the twins had been followed up for six and eight years, respectively, and they all remained healthy. However, the height and weight of the patients with PPB were lower than those of their respective identical sister.

**Conclusions:**

PPB is rare, especially in monozygotic twins. We emphasise the importance of genetic testing and follow-up in monozygotic twins with PPB. During the follow-up, children surviving PPB should be monitored closely for growth and development disorders which caused by chemotherapy.

## Background

Pleuropulmonary blastoma (PPB) is a potentially aggressive, rare childhood neoplasia. It is the most common primary malignant lung tumour in children [1]. PPB is classified into three main types: type I is purely cystic; type II is mixed cystic and solid; and type III is an entirely solid and typically aggressive sarcoma [2]. The type of PPB correlates to the age of diagnosis and prognosis. The 5-year disease-free survival (DFS) and overall survival (OS) rates for Type I PPB are 82 and 91% respectively [3]. For Type II and Type III PPB, the 5-year DFS rate is 59 and 37%, respectively, and the 5-year OS rate is 71 and 53%, respectively [3]. A single-centre report of 41 cases with Type I, II and III PPB in our hospital revealed that the 5-year survival rate was 100, 66.7, and 66.7%, respectively, and the 5-year DFS rate was 100, 66.7 and 55.6%, respectively [4].

PPB has been linked to the mutation of *DICER1* as part of a predisposition syndrome for different types of tumours [5]. PPB is one of the most important causes of *DICER1*-associated morbidity and mortality. While it is uncommon to have more than one individual in a family diagnosed with PPB, some of the other conditions associated with a germline *DICER1* pathogenic variants (e.g., nodular hyperplasia of the thyroid, benign lung cysts) may have a higher penetrance.

Pathogenic germline *DICER1* variants cause a hereditary cancer predisposition syndrome with a variety of manifestations [6]. The risk for most *DICER1*-associated neoplasms is highest in early childhood and decreases in adulthood. Current consensus guidelines for the surveillance of individuals with a *DICER1* pathogenic variant suggest that chest x-ray (CXR) every 6 months from ages 0–7, and then annually from ages 8–12 [7] is vital for improving PPB prevention, surveillance, treatment and follow-up.

## Case presentation

### Case 1

As described previously [8], a girl aged 4 years and 2 months old (Twin1) was diagnosed with PPB Type II. She underwent left lower lobectomy with complete removal (R0 resection) at diagnosis and then completed 12 cycles of chemotherapy with IVADo (ifosfamide, vincristine, actinomycin D, doxorubicin) and IVA (ifosfamide, vincristine, actinomycin D), resulting in cumulative doses of ifosfamide and doxorubicin of 48 g/m^2^ and 240 mg/m^2^. No second surgery was performed. Her older sister (Twin2) developed acute transient hepatitis at about 5 years of age. Their family history showed that their mother and two aunts had thyroid nodules and their maternal grandmother died of thyroid cancer. Analysis of peripheral blood samples revealed a germline *DICER1* mutation: c.C3675A (p.Y1225X) in the twins and their mother [8].

#### Outcome and follow-up

At the most recent follow-up, the twins were about 10 years old and had been followed up for about 74 months. They remained healthy without heart, liver, kidney dysfunction and scoliosis. Twin1’s height and weight were around the 85th and 40th percentile, respectively, and Twin2’s height and weight were around the 92th and 64th percentile, respectively. Both Twin1 and Twin2 remained healthy at their last review. Follow-up imaging to monitor for pulmonary disease will include chest computed tomography (CT) for tumour recurrence and MRI for brain metastasis in Twin1. In Twin1 and Twin2, both of whom had confirmed *DICER1* mutation*,* ultrasound (US) of thyroid and ovaries are recommended every 3 to 5 years and every 6 to 12 months beginning at age 8–10 years, respectively.

### Case 2

**Fig. 1:**
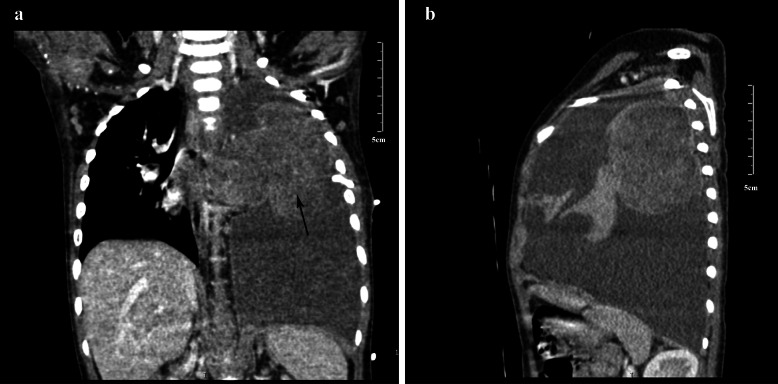
(**a** and **b**) Enhanced chest computed tomography (CT) scan revealed 5.9 × 4.8 × 7.6 cm heterogeneous solid lesion, multiple cystic liquefaction foci can be seen inside

A 19-month-old female baby (Twin3) was referred to our hospital for cough, fever and tachypnoea. A US examination revealed a 9.2 × 10.8 × 16.8 cm liquid zone in the left hemithorax with a 7.6 × 6.9 × 6.1 cm irregular solid lesion inside, and an enhanced chest (CT) scan revealed a 5.9 × 4.8 × 7.6 cm heterogeneous solid lesion with CT value 46 HU, within which multiple cystic liquefaction foci could be observed (Fig. 1).

Twin3 underwent complete resection (R0 resection) at diagnosis, with a large solid tumour measuring 9 × 6 × 5 cm resected from the left lung. Immunohistochemical staining showed Vimentin(+), Ki-67(3%+), EMA(+), NSE(+), Desmin(−), S-100(−), Myo(−), CK(−), SMA(−), SYN(−), CgA(−), CD34(−), CD31(−). Morphological and immunohistochemical features of the resected mass suggested the diagnosis of PPB type III. Abdominal ultrasonography, bone marrow aspiration and cranial MRI detected no metastatic lesions. As PPBs are relatively chemo-sensitive tumours, Twin3 successfully completed 12 cycles of post-operative chemotherapy according to the international PPB chemotherapy regimen IVADo (ifosfamide, vincristine, actinomycin D, doxorubicin) and IVA (ifosfamide, vincristine, actinomycin D). No second surgery was performed.

The identical twins were born with birth weights of 1550 g (Twin 3) and 1450 g (Twin4). Twin3 was diagnosed with bronchopulmonary hypoplasia due to dyspnoea and hospitalised for 27 days in the neonatal department after birth, Twin4 was allergic to mould and she had allergic asthma. Analysis of peripheral blood samples revealed no *DICER1* mutations in these twins.

#### Outcome and follow-up

At the most recent follow-up, the twins were about 9 years old and had been followed up for 99 months. Twin3 was doing well clinically and has remained disease-free for 8 years. Her height and weight were around the 74th and 8th percentile, respectively. Her younger sister (Twin4) had also remained healthy, including with improvement of her allergic asthma. Her height and weight were around the 83th and 12th percentile, respectively. Both twins had no heart, liver, kidney dysfunction and scoliosis. Follow-up imaging to monitor for recurrent pulmonary disease will include chest CT every 3 months in the first year after drug withdrawal, then every 6 months in years 2–4 after drug withdrawal. MRI to check for brain metastasis if had recurrence.

## Discussion and conclusions

Pathogenic germline *DICER1* variants cause a hereditary cancer predisposition syndrome with a variety of manifestations [9]. *DICER1* tumour predisposition (DICER1) is characterised by an increased risk for PPB, thyroid gland neoplasia (multinodular goitre, adenomas and thyroid cancer), ovarian tumours (Sertoli-Leydig cell tumours, gynandroblastoma and sarcoma) and cystic nephroma. PPB occurs primarily in very young children, with a study reporting that Type I PPB becomes evident in infants and young children (median age at diagnosis: 8 months^)^ [10], Type II PPB presents at a median age of 35 months and Type III PPB presents at a median age of 41 months [10]. Sertoli-Leydig cell tumours (SLCT) can occur at any age, most often in adolescents and young adults [7]. Cystic nephroma is most common in children younger than 4 years of age, although *DICER1*-associated cystic nephroma has also occurred in adolescents. Less commonly observed tumours include ciliary body medulloepithelioma (CBME) [7], which are typically identified in young children with an average age at diagnosis of 6 years, pituitary blastoma, which is a rare tumour described in children aged 2 years [7], and embryonal rhabdomyosarcoma (ERMS), which most commonly occurs in pubertal and post-pubertal adolescent girls and young women [7]. The syndrome is caused by mutations in the *DICER1* gene, which is located on the long arm of chromosome 14 in region q32.13; its gene product (DICER1 protein) is a crucial component in the processing of microRNA (miRNA) [11].

Germline loss of function mutations in *DICER1* have been identified in approximately 70–80% of children with PPB [12]. The presence of such germline mutations defines *DICER1* PPB familial tumour predisposition syndrome (FTPS). *DICER1* FTPS increases the risk for multiple tumour types, and the most severe manifestations of pathogenic germline *DICER1* variants tend to present in early childhood, with adulthood characterised by good health [13]. Therefore, the follow-up of patients with PPB is important especially in children, and individuals should also continue to receive appropriate age-and gender-specific preventive screening studies and surveillance.

The DICER1 protein has a central role in development as a switch. Its function is ubiquitous in organogenesis, meaning that all organs are potential targets when this critical protein is faulty [14]. As a developmental switch in the lung, dysembrogenesis results in the formation of cysts which in the lung become PPB Type I. These cysts remain as such or undergo malignant progression to PPB type II or III [14]. The present study involved two pairs of monozygotic twins. Case 1 was twin girls aged 4 years 2 months with *DICER1* mutation, one of whom developed PPB(II) and her identical sibling had acute transient hepatitis. As our previous study reported, whole-genome sequencing of blood samples identified 6,361,526 germline variants in this family (her mother and her sister), including the mutation identified by targeted sequencing (c.C3675A). In addition, the tumour tissue of Twin1 and Twin2 harboured a nonsynonymous variant of *DICER1* (c.G5125A) [15]. However, no *DICER1* mutations were found in Case 2 in the peripheral blood samples of either twin or their mother. Although recent studies have demonstrated that acute loss of *DICER1* leads to liver regeneration inhibition compared to WT littermates [16], and *DICER1* germline mutation has a critical role in liver carcinogenesis [17], the relationship between the *DICER1* gene and acute transient hepatitis is not yet clear. It is important to further explore whether *DICER1* mutations have direct effects on hepatitis.

Inherited diseases are not always expressed in the same way in every individual that carries the same variant in a disease-causing gene. This phenomenon is known as reduced or incomplete penetrance [18]. Variable and incomplete penetrance may explain why clinically healthy individuals can carry potentially pathogenic variants without expressing features of the disease. DICER1 is inherited in an autosomal dominant manner with reduced penetrance. Study reported each child of an individual with a DICER1 germline pathogenic variant has a 50% chance of inheriting the variant, but given the reduced penetrance, many individuals with a germline DICER1 pathogenic variant remain clinically unaffected [19], they may explain why although twin1 and twin2 with DICER mutation, only twin1 developed PPB In our case1. Moreover, cancer is a set of diseases that exhibit not only genetic mutations but also a profoundly distorted epigenetic landscape, the epigenome has a crucial role in the regulation of gene expression, epigenetic changes such as DNA methylome. At the time of the twinning, both twins still have same methylation pattern. Over time, due to intrinsic and environmental factors, the pattern becomes markedly different. The one twin acquires methylation on the promoter site of the gene, which may influence expression and predispose this twin to cancer development [20].

The two PPB patients in the present study were girls. According to a previous study at our hospital [4], and the findings of Knight al et. PPB patients have an equal male-to-female ratio; whether there is no gender difference in monozygotic twins with PPB needs more clinical data support. The two pairs of twins were regularly followed up for 6 and 8 years, respectively and their condition had continued to be stable, but the weight and height of the PPB patients were significantly lower than that of their sister. The toxic effects of chemotherapy on growth are well known, during the phase of chemotherapy children often do not grow. T Moshang Jr. et al. reported that chemotherapy decrease growth velocity and causes growth arrest and bone age retardation and delays catch-up growth. As most of the growth in a child takes place before the age of 4 years and during puberty, it is likely that the effect of chemotherapy on growth will be most pronounced during these periods. One study has suggested that ifosfamide can bind with DNA to form DNA-DNA and DNA-protein cross-links, thus exerting a cytotoxic effect and inhibiting cell proliferation [21]; additionally, doxorubicin will trigger topoisomerase II activity to cause DNA damage [21]; therefore, both affect growth. In vitro studies also have shown doxorubicin and actinomycin D to have a direct effect on growth plate chondrocytes, that in animals resulted in decreased growth and final height [21]. In our study, both patients with PPB were in the growth phase at the time of chemotherapy and had received 12 cycles of chemotherapy with IVADo and IVA, resulting in cumulative doses of ifosfamide and doxorubicin of 48 g/m^2^ and 240 mg/m^2^, respectively. This dose of chemotherapy drugs might have affected the weight and height of the patients with PPB compared with their respective identical sister. Therefore, as the child enters adulthood, continued surveillance for growth and development is necessary.

The two pairs of monozygotic twins in our study were naturally conceived children. A trio-based analysis confirmed that they were monozygotic twins, as they have almost identical single nucleotide polymorphism genotypes (~ 99% identity). Until now, there have been no reports on whether the higher proportion of in vitro fertilisation (IVF) would cause a higher risk of PPB. The question of an increased cancer risk after IVF has been much debated. Spector et al. reported a small, marginally significant association between IVF and overall cancer in childhood and no association between IVF treatment and overall cancer or embryonal tumours [22]. Kaartinen N et al. also reported that the risk of childhood cancer did not appear to be increased in IVF children [23].

Several authors have pointed to a possible correlation between IVF and childhood cancer, mainly of neuroectodermal origin. Petridou et al. indicated the possible association between IVF and increased risk of early onset acute lymphocytic leukaemia (ALL) in children in Greece and Sweden [24], and Hargreave M et al. found that, among children born in Denmark, the use of frozen embryo transfer was associated with a small but statistically significant increased risk of childhood cancer compared to children born to fertile women [25]. A recent study found at most a small, marginally significant association between IVF and overall cancer in childhood in the United States; although this was the largest cohort published to date, this result did not definitively establish an association between IVF and tumours [26]. Therefore, although there may not be a higher risk of PPB arising from the higher proportion of in vitro fertilisation, continued follow-up of children conceived via IVF for cancer occurrence is warranted.

In Types II and III PPB, both systemic chemotherapy and surgical resection are critical components of treatment. Optimal timing for surgery and chemotherapy is dependent upon the type of PPB. Upfront resection is desirable in Type I/Ir tumours, with the decision to append chemotherapy onto treatment. For Type II/III tumours, consideration must be given to the anatomy of the tumour itself: if a tumour is amenable to complete early resection, this approach is preferred. Indeed, up-front complete PPB resection at diagnosis is desirable when possible, but pre-operative chemotherapy may be necessary in instances where this is not possible or presents an unacceptable risk to the patient. For children with huge unresectable tumours, puncture biopsy or surgical biopsy can be performed first. After the lesion has been pathologically confirmed, the size of the tumour can be reduced by four to eight courses of chemotherapy followed by radical surgery [27].

In the present study, as the tumours were amenable to complete early resection at diagnosis, Twin1 underwent left lower lobectomy with complete removal and Twin3 underwent thoracotomy. Both patients received biopsy during the operation. Types II and III PPB require postoperative chemotherapy, and we used the protocol recommended by the International PPB Registry: ifosfamide, vincristine, actinomycin D and doxorubicin (IVADo). The European Cooperative Study Group for Paediatric Rare Tumors (EXPeRT) has identified a threshold of 10 cm as a reasonable cut-off point, above which up-front biopsy followed by neoadjuvant chemotherapy is a reasonable approach. However, no prospective studies examining the timing of resection and chemotherapy exist.

This study reported the long-term follow-up of two pairs of monozygotic twins, now with stable health conditions. *DICER1* testing is recommended for all individuals found to have a PPB of any type and should be performed for all first-degree relatives of patients with *DICER1* mutations, with an emphasis on children under 7 years of age. PPB in one of monozygotic twins is rare, so we will continue to focus on the health condition of these twins closely in the future. Research on the molecular mechanism of PPB in these two cases will also be the subject of our future study.

## Data Availability

All data generated or analysed during this study are included in this published article.

## Abbreviations

PPB: Pleuropulmonary blastoma
CT: Computed tomography
IVAdo: Ifosfamide, vincristine, actinomycin D, doxorubicin
IVA: Ifosfamide, vincristine, actinomycin D
US: Ultrasound
IVF: In vitro fertilisation
